# Hepatic laceration and total parenteral nutrition extravasation due to dislocation of an umbilical venous catheter

**DOI:** 10.1111/jpc.15779

**Published:** 2021-10-27

**Authors:** Francesco Baldo, Angela Pirrone, Antonella Trappan, Laura Travan

**Affiliations:** ^1^ Department of Medicine, Surgery, and Health Sciences University of Trieste Trieste Italy; ^2^ Department of Neonatology Institute for Maternal and Child Health Burlo Garofolo Trieste Italy

## Question

**Fig. 1:**
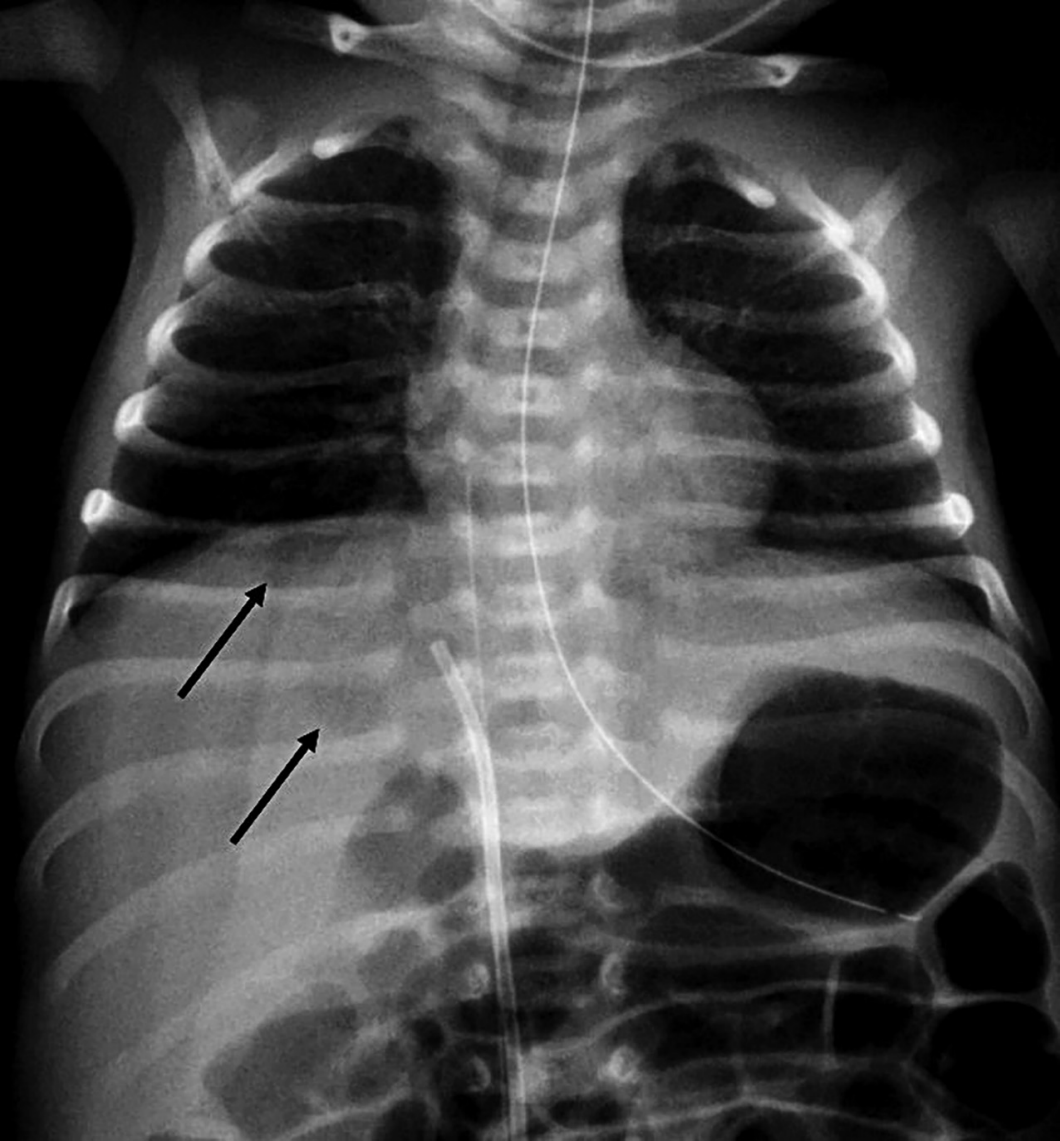
X‐ray image with multiple bubbles in the hepatic area.

**Fig. 2:**
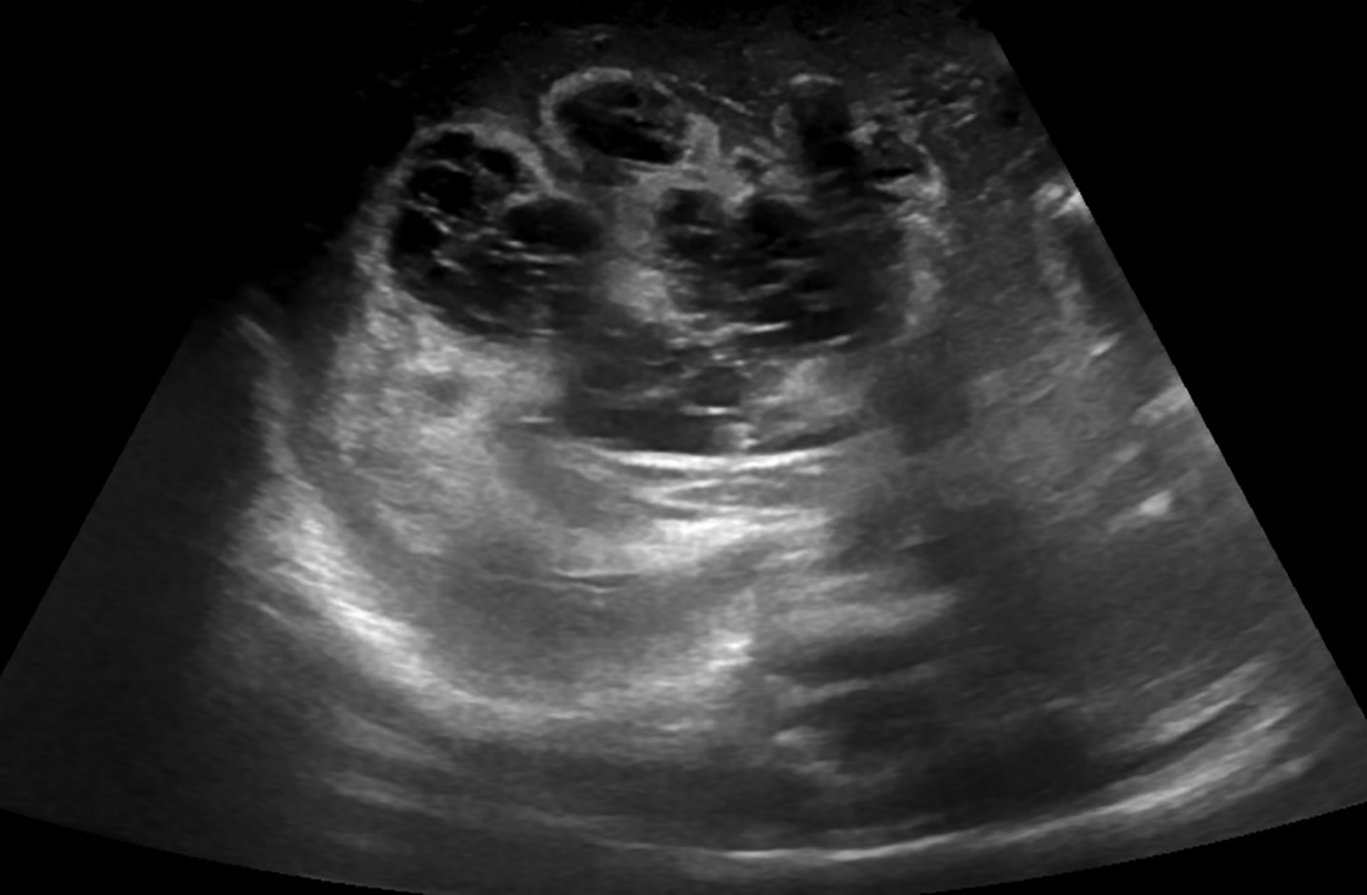
Ultrasound image with the multicystic lesion of the liver's right lobe.

A 32 + 1 week‐old, 1915 g male was born from vaginal delivery after premature rupture of membranes. Apgar index was 8‐9‐10 at 1, 5 and 10 min of life. Due to respiratory distress, he later required non‐invasive respiratory support with nasal Continous Positive Airway Pressure (CPAP). An umbilical venous catheter (UVC) was placed to start parenteral nutrition and antibiotic therapy with ampicillin and tobramycin. Its correct position was confirmed by ultrasound and X‐ray images. Antibiotics were suspended after 36 h, when blood cultures resulted negative. On his fourth day of life, the newborn's general condition declined. His skin became mottled and greyish, his tone and reactivity deteriorated and his abdomen appeared distended: sepsis was suspected. However, C‐reactive protein and procalcitonin resulted negative. An X‐ray image was repeated and highlighted the dislocation of the UVC and the presence of multiple bubbles in the hepatic area (Fig. 1). An abdominal ultrasound showed a voluminous intrahepatic multicystic lesion, extended throughout the entirety of the liver's right lobe, and partially involving the left lobe, measuring 5.2 × 4 × 4 cm and compressing the inferior vena cava (Fig. 2).

What is the most likely diagnosis? (Answer on page 1701)

